# Does higher education enhance public perception of the environment: a quasi-natural experiment based on higher education expansion

**DOI:** 10.3389/fpsyg.2025.1671471

**Published:** 2025-10-07

**Authors:** Hanjin Xie, Fengquan Wu, Jun Li

**Affiliations:** ^1^School of Economics and Management, East China Jiaotong University, Nanchang, China; ^2^School of Public Management, South China Agricultural University, Guangzhou, China

**Keywords:** public environmental perception, higher education, cohort DID, environmental regulation, perception differences

## Abstract

It is now well acknowledged that environmental challenges threaten public health. As knowledge proliferates, public awareness of environmental issues escalates, along with heightened sensitivity to these concerns. This study seeks to utilize the 1999 ‘college entrance examination enlargement’ policy as a quasi-natural experiment. Utilizing data from the 2020 China Family Panel Studies (CFPS), a perception score was established on a scale of 0–10, and a cohort difference-in-differences model was employed to assess the influence of higher education on public environmental perception. The research indicated that advanced education has enhanced the public’s environmental awareness levels. Mechanism studies indicate that higher education enhances environmental awareness by augmenting individuals’ scientific literacy through extended years of study, cultivating favorable views toward the internet, and refining internet usage skills. The enhancement of rational thinking skills via higher education constitutes another avenue of influence. The influence of higher education on improving public environmental awareness demonstrates considerable variability across several aspects. The research demonstrates that voluntary and market-incentive-based environmental restrictions have distinct moderating functions in this process. This research clarifies the fundamental mechanisms of enhanced environmental perception among Chinese inhabitants, offering additional insights into the evolution of environmental consciousness and behavior among the Chinese populace. Moreover, it provides significant insights for policy enhancement, educational philosophy, and urban–rural integration.

## 1 Introduction and literature review

As technology progresses rapidly, environmental challenges are increasingly intertwined with public health outcomes. Researchers Wang et al. (2020) assert that urbanization elevates carbon dioxide emissions, consequently exacerbating environmental degradation in the area. Rodriguez-Loureiro et al. (2021) investigated the correlation between the environment and self-perceived health, revealing considerable heterogeneity based on educational attainment and a robust association between urban green spaces and health disparities, thereby advancing the research perspective on the environment-health nexus. Schleicher et al. (2017) discovered that the absence of environmental elements can result in an inadequate understanding of human well-being and poverty, whereas Turner et al. (2020) contended that air pollution constitutes a worldwide health burden. The study presents epidemiological evidence establishing a correlation between air pollution and cancer incidence and mortality. Researchers, including Suk et al. (2016), who concentrate on pediatric health, discovered that exposure to environmental pollutants during the critical phase of early life development may result in child mortality, indicating that toxic chemical pollution poses a significant threat to their health.

The aforementioned studies indicate that the gravity of environmental issues must not be underestimated, as they are linked to individual health and survival; thus, human decisions in addressing these problems are vital for the future advancement of society. Local governments must cultivate environmental awareness, enhance their regulatory and management capabilities regarding the environment (Yue and Han, 2025), and transparently disclose pollution data to foster public endorsement of renewable energy technologies (Yu et al., 2022). The progression of environmental issues compels the populace to adopt proactive measures for effective response, including engagement in environmental development and its implications for individual health and survival. To respond effectively, individuals should engage in physical activities to enhance health (Quinn and Morgan, 2017) and bolster their environmental awareness (Grogan, 2021), demonstrating a transition from ‘passive’ to ‘proactive’ responses to contemporary environmental issues. The research conducted by Pontes et al. (2024) indicates that an individual’s belief status is profoundly affected by their environment, with environmental perceptions mediating the intention to engage in practical green actions. Intentions to genuinely adopt environmentally friendly practices. Consequently, public environmental perception is an indispensable factor in this change process. Current research on public environmental perception can be categorized as follows: One category involves direct measurement of environmental perception, primarily examining its effects on various societal aspects, including individual environmental behavior and mental health. Another category focuses on public concern for the environment and environmental awareness, with the research process serving as an extension of public environmental perception. A third category provides a specific analysis of environmental pollution, reflecting the impact mechanisms of public environmental perception within the study. A distinct group delineates environmental contamination and examines the mechanisms influencing public environmental perception inside the research.

However, it must be noted that both environmental concern and environmental awareness are concepts that place greater emphasis on the individual’s ‘proactivity.’ This is evident from the variables selected in academic research on the subject (Li et al., 2022; Mahi, 2024). Their research highlights the cognitive and behavioral outcomes resulting from individuals actively engaging with environmental information. However, this ‘proactive’ cognitive and behavioral aspect is not the primary focus of this study. This is because, even if an individual has not received higher education, their family or social environment is more likely to influence their proactive environmental behavior, which aligns with the definitions of environmental concern or environmental awareness as defined in academic research. In contrast, the environmental perception discussed in this study holds significant implications for higher education. Environmental perception exhibits a more ‘passive’ nature, manifesting as an instinctive response, which, as higher education cultivates higher-level qualities and cognitive abilities, leads to more sophisticated instinctive responses. Furthermore, an individual’s concern for the environment or possession of environmental awareness necessarily implies that they possess environmental perception, but the reverse is not true. This not only serves as the initial motivation for this study but also underscores its theoretical significance in linking environmental perception to higher education. The research process itself imbues an understanding of environmental behavior with deeper educational implications.

This paper will examine existing studies from the subsequent angles. First, research on public environmental perception has established that it encompasses not only an individual’s awareness, cognition, and comprehension of information regarding their environment but also serves as a crucial psychological foundation that affects public behavioral decisions and environmental protection consciousness. Numerous scholars have asserted that environmental perception influences public environmental behavior (Barr, 2003; Carfora et al., 2017; Gholamzadehmir et al., 2019). Additionally, Wang et al. (2023) noted that environmental perception affects an individual’s subjective well-being through community attachment. Furthermore, Neo et al. (2017) comprehensively demonstrated the impact of environmental perception on individual behavioral choices, utilizing Malaysian urban residents as subjects. The study confirmed the role of individual psychological factors in behavior, highlighting that shared psychological decisions can serve as a foundational reference for the design of sustainable development programs. Simultaneously, environmental perception may also illustrate the impact of elements such as socio-cultural influences, economic status, and education on individual viewpoints, resulting in variations in environmental views among distinct social groups. Bjørndal et al. (2023) asserted that residents’ perception of noise and pollution correlates with a significantly increased likelihood of mental illness symptoms, highlighting the critical role of social environment and residential area characteristics in influencing population mental health.

The aforementioned scholars’ investigations into “environmental concern” and “environmental awareness” fundamentally derive from the expansion of “environmental perception.” Regarding ‘environmental pollution,’ which directly influences public environmental perception, scholars primarily concentrate on its characteristics and management. The growth of related study has a lengthy history; nonetheless, it serves as an indirect manifestation of public environmental perception. Secondly, Wu et al. (2020) asserted that, amid the decline in environmental quality, governmental environmental governance and public engagement in environmental management have emerged as the paramount factors. Zhang and Zhang (2024) investigated the correlation between governmental environmental governance and regional green growth, concluding that such governance fosters innovation through the facilitation of the ‘innovation compensation effect’. The government’s actions partially mirror the public’s ecological needs shaped by environmental perceptions, and research indicates that residents’ environmental perceptions influence other factors. Liu et al. (2023) demonstrated, utilizing data from CEOs of publicly traded companies between 2011 and 2020, that CEOs influence the advancement of social and environmental responsibility, with variations in their educational attainment affecting this influential capacity. The aforementioned studies have demonstrated the inherent connection between environmental perception and pollution. Consequently, examining public environmental perception aids in comprehending current public attitudes toward environmental governance and contributes to the refinement of environmental policies and the enhancement of governance efficacy.

It can be concluded that current research emphasizes two distinct aspects: the ‘environment,’ which encompasses studies on pollution emissions, energy consumption, and health impacts, and ‘environmental perception,’ which involves analyzing specific issues through the lens of public or governmental perceptions of the environment. Conversely, it emphasizes ‘environmental perception,’ which entails utilizing the degree of public or governmental awareness of the environment to perform comprehensive assessments of an issue or employing ‘environmental perception’ to enhance the study’s content. These foundations indicate that public environmental perception research is more acknowledged and that the public’s capacity to sense the environment is being enhanced as urbanization in China advances. Nevertheless, this paper identifies certain deficiencies. Primarily, the majority of pertinent studies consider ‘environmental perception’ as a ‘cause’ and have yielded significant outcomes across several domains, whereas limited research treats it as an ‘result’ and investigates its own ‘consequences’ in the literature. There is scant literature regarding its formation and development as an effect; similarly, environmental pollution is not the sole determinant of environmental awareness. Furthermore, the correlation between the environment and self-perceived health varies by educational level, as noted in prior studies, indicating that the enhancement of public environmental perception is not attributable to a singular factor. Secondly, the research approach is somewhat simplistic, mostly depending on surveys and interviews to encapsulate the findings and inadequately employing econometric techniques to analyze the determinants of public environmental perception. Thirdly, in addition to the state of the environment, they overlooked the significance of higher education in personal development and societal transformation, as well as the relationship between its impact and environmental change. All the aforementioned difficulties require thorough examination.

This research seeks to experimentally investigate the long-term effects of higher education on public environmental perceptions, utilizing the 2020 China Family Panel Studies (CFPS) data and a cohort difference-in-differences model, while also analyzing the potential mechanisms involved. The incremental contributions of this study are as follows: This research presents new empirical evidence about the substantial enhancement of people’s environmental awareness levels within the framework of long-term development. Education has impacted numerous facets of society, the economy, and demographics. Current studies indicate that education diminishes the overall crime rate within society (Machin et al., 2011), and variations in educational participation rates directly influence future income inequalities among individuals (Huang et al., 2022). Furthermore, the increase in college entrance exam enrollment has markedly impacted intergenerational mobility between urban and rural populations (Duan et al., 2022). Consequently, higher education unequivocally impacts the development of the public’s environmental perception. This research examines the effect of higher education in enhancing the environmental awareness of Chinese citizens and its mechanisms of influence. This research examines higher education concepts and methodologies, integrating the disparities in environmental policy requirements between urban and rural regions for theoretical analysis. This research offers an educational elucidation at the individual level on the phenomena of varying environmental policy requirements stemming from urban–rural growth discrepancies. It focuses on the influence of higher education on individual distinctiveness, providing insights for environmental policy development and strategies for fostering public collaboration. The research method enhances our thorough comprehension of the roles and significance of higher education. This study, conducted within a constructivist framework, elucidates the fundamental reasons of changes in people’ environmental views amid China’s urbanization, consequently providing insights into remedies for China’s environmental and associated livelihood challenges.

## 2 Theoretical hypothesis

### 2.1 Higher education for public environmental perception

Since the establishment of New China and through the reform and opening-up period, China has undergone significant social transformations in a remarkably brief timeframe, swiftly evolving from a traditional agrarian society to an industrialized nation, and is currently progressing toward modernization with the advancement of innovative productive forces. The swiftly evolving development process will undoubtedly affect both the material and spiritual dimensions of the Chinese populace, influencing their perceptual capacities across many domains. Current theories indicate that the public’s perception is shaped by the prevailing social environment, encompassing not only the physical living conditions but also the intangible aspects such as humanitarian considerations and the cultural ambiance. The discovery that individuals with diminished self-esteem exhibit greater social activity than those with elevated self-esteem, amassing numerous online friends as a means of compensating for their low self-worth, is derived from a study of Facebook users, which substantiates the notion of socially compensatory friendships within the online environment (Lee et al., 2012). Moreover, a substantial correlation exists between the public self-consciousness and social anxiety of automobile and taxi drivers, which may indirectly contribute to their aberrant driving behavior, with notable gender disparities in this relationship (Huang et al., 2018). Moreover, ‘food safety self-protection awareness’ arises from the combined influences of recurrent encounters with food safety issues and societal advancements that enhance the public’s perceived capacity to invest in health (Wang et al., 2020).

The environmental pressure generated by a country’s economic activities and its environmental carrying capacity determines the sustainability of the country’s social and economic development (Atanu, 2022), and while the regional economy is developing rapidly, it is often facing deteriorating resource and environmental pressures due to irrational industrial structure and insufficient environmental protection investment (Xu and Tan, 2020). In addition, the link between regional development and environmental change is difficult to portray using a simple linear relationship, and urbanization development and resource and environmental levels may constantly show recurring relationships of coupling and decoupling, which reflects the dynamic evolutionary characteristics of the factors associated with environmental change, and the complex and unique nature of the process of examining the development of Chinese residents’ environmental perceptions. This paper is concerned that the level of public perception of the environment has become prominent, and is committed to observing the formation process of environmental perception of contemporary Chinese residents, and providing possible explanations and inferences for its evolutionary development and changing characteristics. Specifically, this paper will analyze the role of higher education in the enhancement of public environmental perception in China from the individual and social perspectives, based on the macro context of urbanization and development.

Existing studies indicate that public environmental perception is shaped by environmental pollution levels and individual education, along with factors such as social media information, local pollution levels (Liu et al., 2021; Cong et al., 2021), the behavioral attitudes of role models, and health status (Zerback and Peter, 2018; Ghirga, 2024). The regional government’s waste separation strategy and various initiatives aimed at preventing and controlling air, water, and soil pollution will directly induce a shift toward environmentally conscious behavior among residents. This shift represents both the development of public environmental awareness and a tangible reflection of the public’s environmental perceptions. When green policies yield substantial positive environmental outcomes, the probability of public adherence to sustainable behaviors increases; conversely, if the effects of environmental policies are negligible, public engagement diminishes. Simultaneously, those with advanced education possess a more profound comprehension of environmental degradation and non-ecological practices, are more inclined to exhibit environmental concern, and thus are more amenable to endorsing governmental environmental regulatory laws. Furthermore, the influence of an individual’s physiological health status is paramount. The Global Burden of Disease Report 2017, published by the World Health Organization (WHO), indicates that for every 1 million deaths in China, 1,611 are attributable to air pollution. This underscores that a significant number of individuals in China endure respiratory and cardiovascular ailments due to environmental pollution, and the environmental perceptions of this demographic tend to be more extreme.

The proliferation of the college entrance examination has converted China’s education system from elitist to mainstream education. As the availability of higher education possibilities expands, an increasing number of individuals are pursuing higher education, resulting in a rise in the average years of schooling for the overall population of China. Historically, the dissemination of environmental policies promoting sustainable behaviors faced challenges due to the generally low literacy rates among the populace. However, with the proliferation of college entrance examinations, individuals with higher education are now more adept at recognizing the perils of environmental degradation, leading to an enhanced awareness of ecological issues. Significantly, as the progeny of individuals lacking higher education attain such qualifications, their participation in economic endeavors progressively surpasses that of their parents, thereby amplifying their influence within the family. This facilitates the promotion and dissemination of the offspring’s ‘environmental discourse’ within the familial context. The collective environmental awareness of society is progressively enhanced by a confluence of elements.

It is essential to acknowledge that China’s educational advancement, alongside the extension of schooling duration and the expansion of educational opportunities for its populace, has also revealed structural educational disparities stemming from the urban–rural divide (Zahl-Thanem and Rye, 2024). Consequently, it is necessary to examine the urban–rural variations in higher education, as these may influence the explanatory power regarding higher education’s impact on public environmental perceptions. Simultaneously, variations in work environments directly influence individual environmental perceptions. Ruepert et al. (2017) asserted that companies must consider their environmental context when promoting eco-friendly behaviors among employees, as this affects the employees’ environmental responsibility. Moreover, regional healthcare resources are intrinsically connected to citizens’ access to healthcare, perhaps resulting in variations in how higher education affects public environmental perceptions. This study suggests Hypothesis 1 based on the preceding analyses.

*Hypothesis 1*: Holding other factors constant, higher education is conducive to promoting public environmental perceptions, and there is heterogeneity in this effect between urban and rural areas, the degree of environmental pollution, and medical resources.

### 2.2 Mechanisms by which higher education influences public perceptions of the environment

#### 2.2.1 Scientific and cognitive mechanisms for the promotion of years of education

Peña-Ayala and Villegas-Berumen (2020) asserted that college education is a crucial phase in fostering personal development. Schwerter et al. (2022) investigated the factors influencing students’ test performance, specifically evaluating whether supplementary e-learning practice opportunities could enhance the performance of contemporary university students. The findings indicate that practice within a higher education context can substantially improve student performance and also highlight the widespread acknowledgment of fundamental competencies, such as individual cognitive talents, in higher education. Higher education encompasses a diverse array of fundamental science courses, including physics, chemistry, and biology for science and engineering majors, alongside elective general education courses. These offerings enable students to comprehend the composition of matter and the processes of change, which are crucial for understanding environmental chemical pollution and energy conversion.

Simultaneously, students of both science and engineering and literature can experience the interdisciplinary learning and research fostered by higher education. In this environment, individuals can not only amass scientific knowledge but also deepen their comprehensive understanding of intricate scientific phenomena. The pursuit of avant-garde knowledge in higher education enhances students’ receptivity to new ideas and equips them to make informed judgments regarding future developments based on these phenomena. Consequently, the aforementioned factors contribute to the advancement of future environmental perception among the educated populace, whose qualifications have been enhanced and whose scientific cognitive abilities have also been augmented, thereby impacting the evolution of future environmental awareness. Consequently, this paper posits hypothesis 2a.

*Hypothesis 2a*: Holding other factors constant, tertiary education improves the public’s scientific awareness and thus contributes to the public’s environmental perception.

#### 2.2.2 Mechanisms for access to information in higher education training

Higher education not only enhances the scientific cognitive skills of university students but also considerably improves their capacity to utilize scientific tools to facilitate their learning. Karunarathne and Calma (2023) asserted that the significance of higher education in cultivating creative thinking skills has been extensively acknowledged. Their study evaluated knowledge creation and problem-solving through three principal themes, revealing that higher education’s improvement of students’ competencies in these domains can benefit them in any professional field. The advancement and proliferation of online technologies render digital learning essential for higher education, necessitating ongoing innovation and research for effective integration. In their research, Aler Tubella et al. (2023) examined the pedagogical approaches for AI in higher education. Their interviews with 11 experts across five countries identified the strategic programs necessary for AI integration and the resources required to support students across various disciplines.

This indicates that pedagogical techniques in higher education focus on the latest technical theories and scientific methodologies, hence enabling students to comprehend advanced theoretical concepts and proficiently utilize scientific instruments. Furthermore, a body of literature investigates the effects of Internet usage among university students, including the ramifications of problematic Internet use (Sánchez-Fernández et al., 2023), the influence of Internet use on students’ well-being (Adorjan et al., 2020), and a study exploring the correlation between Internet use and mental illness (Kitazawa et al., 2018). While these studies predominantly address the adverse consequences of Internet use, they concurrently affirm the pivotal role of higher education in enhancing individuals’ Internet proficiency. Consequently, this paper posits Hypothesis 2b.

*Hypothesis 2b*: Holding other factors constant, tertiary education improves the public’s ability to use the Internet to access information, thus contributing to the public’s perception of the environment.

#### 2.2.3 Mechanisms of rational thinking fostered by higher education

Higher education possesses the capacity to cultivate students’ competencies across multiple dimensions, and while the academic environment augments individuals’ scientific comprehension, its pedagogical framework is equally crucial for the advancement of rational thought. Researchers Tetzlaff et al. (2022) have examined personalized instruction in the classroom as a situation in which the higher education landscape embodies a logical educational framework. Researchers, including Shahzad et al. (2024), investigate the perception of artificial intelligence technology in higher education, revealing that trust is fundamental, indicating that higher education emphasizes the cultivation of logical thinking in students. Daniel and Watermann (2018) developed a quasi-experimental test of rational choice theory, grounded in higher education programs, demonstrating that students’ decisions, influenced by perceived benefits, were informed by a degree of rational thought. Gabay-Egozi Shavit et al. (2009) contended that the rational choice theory of education perceives students’ educational decision-making as a binary selection among various alternatives that promise long-term benefits while minimizing the risk of immediate failure, thereby enabling individuals impacted by higher education to make a rational choice among these options. Individuals impacted by higher education might enhance their rational thinking during the learning process. Consequently, Hypothesis 2c is proposed.

*Hypothesis 2c*: Holding other factors constant, higher education increases the level of rational thinking of the public, thus contributing to the public’s environmental perception.

## 3 Data sources and empirical strategies

### 3.1 Data sources and processing

The microdata presented in this research is sourced from the China Family Panel Studies (CFPS). Launched by the Centre for Social Science Research at Peking University, the survey is a nationwide, comprehensive social tracking initiative designed to capture the transformations in China’s society, economy, demography, education, and health by gathering data at the individual, household, and community levels. The CFPS emphasizes both the economic and non-economic well-being of China’s residents, aligning closely with the primary focus of this paper’s research. In 2010, the CFPS conducted a baseline survey throughout 25 provinces, municipalities, and autonomous regions in China. In 2010, CFPS executed a baseline survey throughout 25 provinces, municipalities, and autonomous regions of China, establishing the permanent tracking population for CFPS surveys, which are conducted biennially. The CFPS offers data on individuals’ birth year, educational attainment, parenting status, and Internet usage frequency, alongside survey responses regarding various social issues, including the environment, employment, healthcare, and education, thereby fulfilling the analytical requirements of this paper.

This paper utilizes the CFPS 2020 survey data, primarily employing the adult questionnaire personal database as the main data file. It integrates the family economic database, family relationship database, personal core variable database, and the child proxy database, which encompasses children’s information from CFPS 2020. This integration is conducted by the standardized CFPS identifiers, including the personal code, personal intra-household code, and family sample code, to compile the CFPS 2020 dataset. Matching is conducted to acquire the CFPS 2020 summary data, encompassing individual and household information of the sample. This paper downloads official statistical yearbooks from the National Bureau of Statistics and provincial statistical bureaus to control the characteristics of the sample’s region during analysis, selects the necessary urban construction and development variables, and integrates them based on the provincial information published in the CFPS. The CFPS questionnaire simultaneously documented various categories of ‘abnormal responses’ (e.g., −10, −9, −8, −2, −1 denote unable to judge, missing, not applicable, refused to answer, and do not know, respectively). This paper excludes samples containing these responses based on the specified classifications, ultimately yielding a cross-section that encompasses aggregated data on individuals, families, and social information, resulting in a total sample size of 16,043.

### 3.2 Empirical strategy

This paper aims to integrate the attributes of existing data and evaluate the causal relationship between higher education and the improvement of public environmental perception. It draws on established literature (Duflo, 2001) and employs the cohort difference-in-difference method to ascertain the causal impact of the college entrance examination expansion policy on public environmental perception. The model is structured as follows:


(1) Eawareipc=α0+α1Cohortc×edup+β1Xi+β2Xp×Cohortc+γc+πp+εipc

In the aforementioned equation, the subscript i signifies the individual, p represents the province of residence, and c indicates the birth cohort. The dependent variable Eawareipc denotes the individual’s degree of environmental perception, assessed by the 2020 CFPS questionnaire, ‘Overall, how serious do you think environmental problems are in our country?’ This inquiry is quantified by responding to a scale from 0 to 10, where 0 signifies not serious and 10 denotes highly serious. The definition of the dependent variable in this study differs significantly from other environmental concern or environmental awareness variables, as it places greater emphasis on the passivity of individuals in relation to environmental impacts. The primary explanatory variable of the college entrance examination expansion policy is designated as an interaction term of Cohortc and edup , where Cohortc indicates whether an individual belongs to the cohort impacted by the policy, and edup denotes the magnitude of the provincial expansion. Individuals typically undertake the college entrance examination at 18 years of age; thus, those under 18 during the 1999 expansion of the examination are classified as having experienced the ‘college entrance examination expansion’ policy and are designated as the post-intervention population, specifically those born after 1981, receiving a value of 1 in the cohort. Conversely, individuals over 18 in 1999 are deemed not to have experienced the ‘college entrance examination expansion’ and are classified as the pre-intervention population, namely those born before 1981, receiving a value of 0 in the cohort.

The expansion variable indicates that, while the growth of colleges and universities is a national policy, local institutions primarily implement the expansion of the college entrance examination. Consequently, the more resources available to colleges and universities, the higher the probability that high school graduates will benefit from this policy expansion (Tam and Jiang, 2015). Therefore, the expansion variable must account for provincial disparities in the college entrance examination’s expansion to some extent. Consequently, the variable representing the degree of expansion must partially encapsulate the interprovincial disparities in enrollment growth. Accordingly, this paper designates the expansion degree variable edup as ‘the ratio of students enrolled in regular colleges and universities to those enrolled in adult colleges and universities within each province’.

Lü and Zhang (2019) developed indicators to assess the extent of enrollment expansion by examining the number of students enrolled in local colleges and universities. These indicators not only fulfill this requirement but also take into account the significance of adult higher education as a crucial component of China’s higher education system, thereby incorporating the enrollment figures from adult higher education institutions into their analysis. The enrollment of students in adult higher education institutions is a significant aspect of China’s higher education system, reflecting the extent to which expansion policies have altered the distribution of opportunities among various student demographics, about environmental influences. Regarding control variables, *X*_i_ represents individual characteristics, encompassing gender and hukou status; *X*_p_ denotes provincial characteristics from 1998, including economic status and various factors influencing individual environmental perception, primarily per capita GDP, employment in the education sector, average worker wages, employment in environmental and public facilities management, and the number of health institutions. Additionally, it includes industrial wastewater emissions, industrial sulfur dioxide emissions, industrial soot emissions, and average concentration of inhalable particulate matter. These control variables interact with Cohort_c_ to allow the impact of the birth cohort to vary according to specified provincial characteristics, thereby capturing the influence of provincial attributes. γc represents Individual Birth Cohort Fixed Effects; πp is Province Fixed Effects; and εipc is a random perturbation term.

Table 1 presents the assignment techniques and descriptive statistics of the variables. The table reveals that the mean value of the environmental perception variable is 6.255, suggesting that the public generally acknowledges the existence of environmental issues in society. Additionally, the substantial standard deviation of the college entrance examination expansion variable indicates significant disparities in higher education resources across provinces, thereby corroborating the validity of the prior analysis presented in this paper.

**Table 1:** Assignment of variables and descriptive statistics.

Variable designation	Variable assignment method	Number of observations	Mean	Standard deviation
Environmental Perception	The value range is [0, 10], where higher values signify a greater assessment of the severity of environmental issues and an elevated level of environmental perception.	16,043	6.255	2.843
Policy for Expansion of Enrollment in the College Entrance Examination	Interaction variable between the impact of the policy of expanding enrollment in the college entrance examination and the degree of expansion.	16,043	0.487	2.891
Degree of Enrollment Expansion	Proportion of students enrolled in general colleges and universities compared to those enrolled in adult colleges and universities, categorized by province, 1998.	16,043	1.827	6.496
Gender	Male = 1; Female = 0.	16,043	0.495	0.5
Hukou status	Agricultural household = 1; Non-agricultural household = 3; Resident household = 5.	16,043	2.163	2.732
Provincial GDP per capita	Logarithmic provincial GDP per capita, 1998.	16,043	8.622	0.844
Number of Employees in the Education Sector in the Province	Employment figures in the education industry by province, 1998, presented in logarithmic terms.	16,043	1.628	0.731
Average Wage of Workers in Provinces	Logarithmic mean earnings of laborers in the provinces in 1998.	16,043	8.814	0.316
Number of Employees in Environment and Public Facilities Management	Number of people employed in the environment and utilities management industry by province, 1998, in logarithmic terms.	16,043	−0.631	1.054
Quantity of healthcare facilities in the province	Number of health institutions by province, 1998, in logarithmic terms.	16,043	6.433	0.873
Discharge of industrial wastewater at the provincial level	Industrial wastewater discharges by province, 1998, in logarithms.	16,043	8.420	1.346
Emissions of sulfur dioxide from industrial sources	Industrial sulfur dioxide emissions by province, 1998, in logarithms.	16,043	10.454	1.444
Provincial emissions of industrial soot	Logarithmic industrial soot emissions by province, 1998.	16,043	9.857	1.261
Average concentration of inhalable particulate matter	Logarithmic average concentrations of respirable fine particulate matter by province, 1998	16,043	4.257	0.835

## 4 Empirical results and analyses

### 4.1 Benchmark regression results

This study analyzes the impact of higher education on public environmental perception, as indicated by Equation 1, with regression findings presented in Table 2. Column (1) presents the regression outcome without the inclusion of control variables, indicating that more education substantially enhances the public’s environmental perception. This paper accounts for individual characteristics that may influence public environmental perception, with the regression analysis results presented in column (2) of Table 2, indicating that higher education enhances public environmental perception. Moreover, taking into account the provincial disparities in higher education, significant variations exist in economic development, infrastructure, and educational attainment across provinces, potentially resulting in divergent environmental perceptions among individuals from different regions. Consequently, column (3) additionally accounts for the influence of provincial features beyond the control of individual characteristic components. The findings indicate that the coefficient for the variable representing higher education is 0.183 and is statistically significant at the 1% level. The aforementioned data indicate that greater education considerably enhances the public’s environmental perception. The analysis results indicate that higher education can promote the improvement of public environmental awareness by 1.58–1.83%. It must be acknowledged that the *R*^2^ values of the regression results in columns (1) to (3) of Table 2 are relatively low, but this is since this study controlled for a large number of individual and provincial-level fixed effects in the regression, which is a normal situation in multiple regression analysis.

**Table 2:** Impact of higher education on public perception of the environment.

Variables	(1) Environmental Perception	(2) Environmental Perception	(3) Environmental Perception
Experience of expansion × degree of expansion	0.158*** (0.047)	0.163*** (0.047)	0.183*** (0.050)
Gender		0.112*** (0.044)	0.111** (0.044)
Hukou status		0.038*** (0.008)	0.038*** (0.008)
Provincial GDP per capita × experience of expansion			−0.226** (0.112)
Number of employees in the education sector in the province × experience of expansion			−0.053 (0.131)
Average wage of workers in the province × experience of expansion			0.272 (0.202)
Number of employees in the environment and public facilities management industry in the province × expansion experience			−0.021 (0.100)
Number of health organizations in the province × experience expansion			0.062 (0.091)
Industrial wastewater emissions in provinces × expansion of experience			0.149** (0.068)
Sulfur dioxide emissions from industry in provinces × expansion of experience			0.034 (0.066)
Emission of industrial soot in provinces × expansion			−0.043 (0.069)
Average concentration of respirable fine particulate matter in province × experience expansion			0.082 (0.054)
Birth cohort fixed effects.	Yes	Yes	Yes
Province fixed effects	Yes	Yes	Yes
Observation	16,043	16,043	16,043
*R* ^2^	0.069	0.070	0.071

**, ***denote significance at the 5%, 1% levels, respectively, with standard errors provided in parentheses.

### 4.2 Parallel trend test

This paper employs the cohort difference-in-differences methodology to examine the long-term effects of higher education on public environmental perception. A critical assumption of this method is the parallel trend assumption: in the absence of policy effects on the treatment group, the trends of the outcome variables for both the treatment and control groups should remain consistent without systematic disparities. In this research, it is posited that in the absence of the gaokao expansion policy, the environmental perceptions of persons who experienced the gaokao expansion and those who did not should exhibit uniformity across all regions, devoid of systematic disparities. Consequently, this research references prior studies and employs Equation 2 to do a parallel trend test, designating individuals aged 18 in the year the gaokao expansion policy was enacted (namely, the birth cohort of 1981) as the benchmark group. This study examines the interaction between the dummy variables of the 12–17-year-old cohort and the 19–29-year-old cohort with the expansion degree variable to perform the parallel trend test.


(2) $$\text{Eawareipc}=\eta 0+\sum_{12,n\neq 18}^{29}\mathrm{\delta n}(\text{An}\times \text{edup})+\eta 1X1+\eta 2X2\times \text{Cohortc}+\mathrm{\gamma c}+\mathrm{\pi p}+\text{εipc}$$

**Figure 1:**
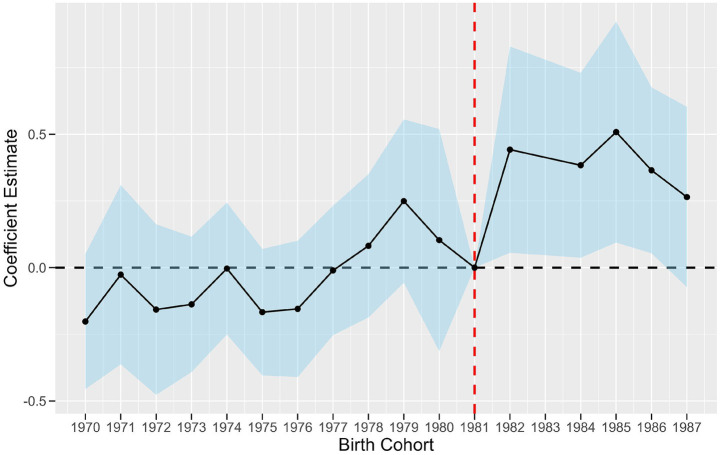
Impact of the HEI expansion policy on public perception of the environment. Birth cohorts 1970–1980 are unaffected individuals aged 19–29 in the year the policy was implemented, and birth cohorts 1982–1987 are affected individuals aged 12–17 in the year the policy was implemented; the coefficient significance interval is 95 percent.

*A*_n_ represents a series of age dummy variables, denoting whether the individual’s age at the implementation of the college entrance examination expansion policy is n; it assumes a value of 1 if true and 0 otherwise. The individual aged 18 in that year serves as the benchmark group for this study and is excluded from Equation 2. The interpretations of the other variables remain consistent with those in Equation 1 of this paper. This paper conducts a parallel trend test, with the results of the regression coefficient δn illustrated in Figure 1. The red vertical line in Figure 1 represents the baseline group cohort of 1981, while the blue area encompassing the line indicates the 95% confidence interval of the coefficient estimates. Figure 1 illustrates that for the birth cohort before 1981, the 95% confidence interval of the interaction term’s coefficient encompasses zero, indicating that the coefficient estimates oscillate around zero. This suggests that before the implementation of the college entrance exam expansion policy, regions with varying levels of higher education influence do not exhibit heterogeneous cohort trends, thereby reinforcing the parallel trend assumption of this study. Conversely, for the birth cohort from 1981 onwards, the interaction term’s coefficient begins to rise progressively. Consequently, it can be concluded that none of the regression coefficients for individuals aged 19–29 who did not benefit from the higher education expansion policy are significant, whereas the regression coefficients for individuals aged 12–17 who were impacted by the policy are significantly positive. The aforementioned research indicates that higher education influences individuals’ environmental perception.

### 4.3 Robustness test

#### 4.3.1 Placebo testing

The improvement of public environmental awareness is not just attributable to increased education; it may also result from many random circumstances. To eliminate this possibility, this research does a placebo test using the following two methods. Initially, a virtual Gao Kao expansion policy is devised for study, utilizing the pre-1981 birth cohort that remained unaffected by the 1999 Gao Kao expansion policy. This research posits that the gaokao expansion policy is enacted 2 and 3 years earlier, respectively, and evaluates whether the actual effects of the gaokao expansion policy will be muddled at these intervals based on the test results. Secondly, randomly interchange the values of the degree of expansion across 31 provinces, interact the resultant virtual degree of expansion variable with the variable indicating whether or not expansion occurred, re-estimate the benchmark model, conduct the replacement test 1,000 times, and analyze the test outcomes.

Initially, imagine that the college entrance examination expansion policy was enacted in 1997. The impacted birth cohort is 1979–1980, specifically persons aged 17–18 in 1997, and this segment of the dummy beneficiary cohort variable is designated a value of 1. Consequently, persons born in 1978 and earlier constitute the unaffected birth cohort, and this segment of the dummy beneficiary cohort variable is assigned a value of 0. Secondly, assuming the college entrance examination policy expansion was enacted in 1996, the impacted birth cohort comprises individuals aged 16–18 born between 1978 and 1980, designated with a value of 1 for the dummy beneficiary cohort variable. Conversely, individuals born in 1977 or earlier belong to the unaffected birth cohort, receiving a value of 0 for the same variable. In a manner akin to the benchmark regression, this analysis posits that the gaokao expansion policy was enacted in 1997 and 1996, respectively. The study interacts the dummy beneficiary cohort variable with the degree of expansion variable to derive the pseudo gaokao expansion policy variable, thereby capturing the policy effect. The regression results are displayed in Columns (1) and (2) of Table 3, with Column (1) illustrating the outcomes of the regression assuming a policy implementation 2 years earlier, in 1997, and Column (2) detailing the results for a three-year earlier implementation, in 1996. The results show that the hypothetical 1997 and hypothetical 1996 high school expansion policies do not have a significant effect on the public’s perception of the environment.

**Table 3:** Robustness test: placebo test.

Variables	Dependent variable: environmental perception	
	(1) Assuming the implementation of the Gao Kao expansion policy in 1997	(2) Assuming the implementation of the Gao Kao expansion policy in 1996
Virtual beneficiary cohort × Degree of expansion	0.156 (0.147)	0.167 (0.115)
Control variables	Yes	Yes
Birth cohort fixed effects	Yes	Yes
Province fixed effects	Yes	Yes
Observation	10,968	10,968
*R* ^2^	0.049	0.049

**Figure 2:**
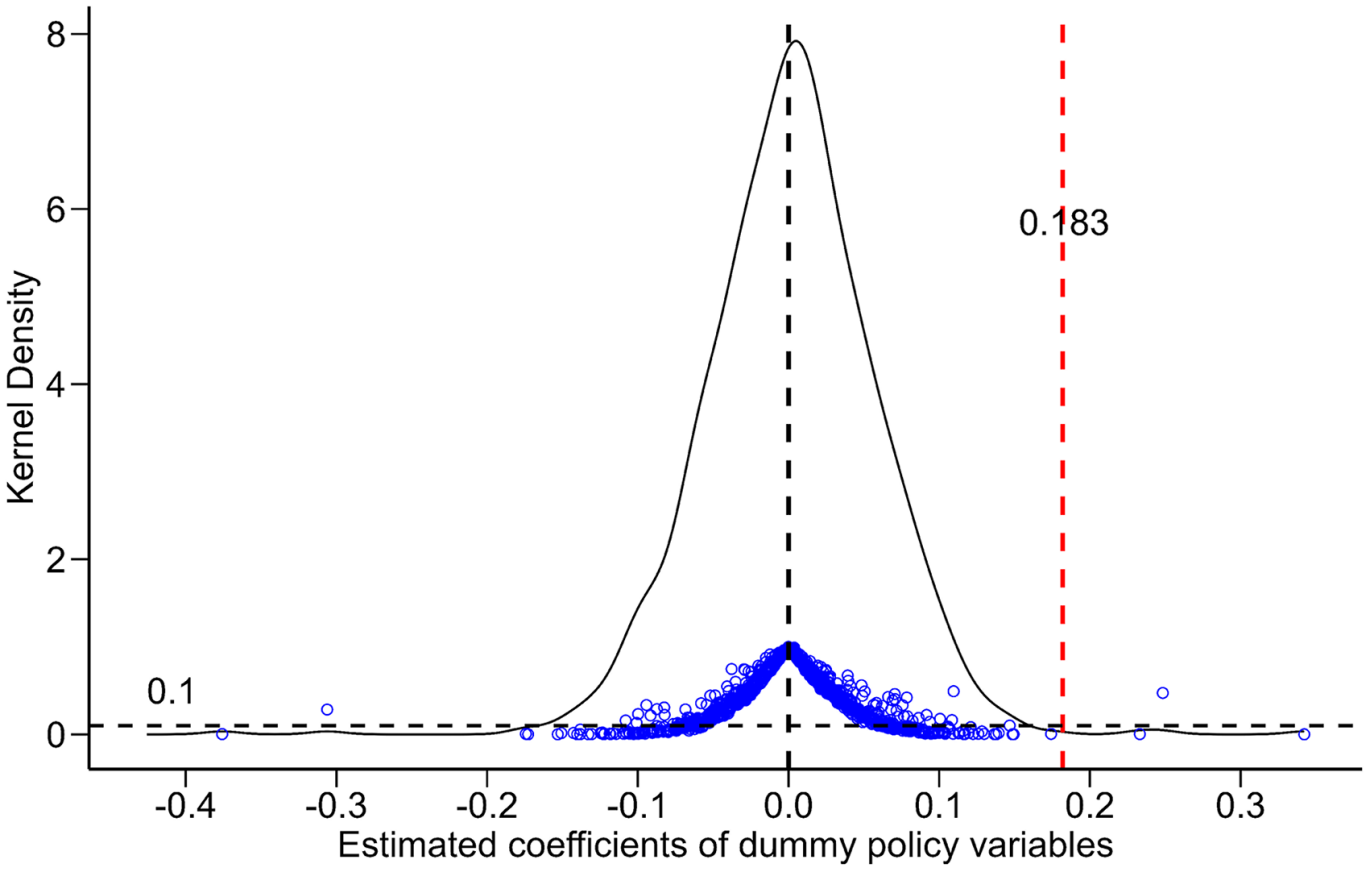
Placebo test results.

The second placebo test method illustrates the distribution of the estimated coefficients in Figure 2. The horizontal axis denotes the magnitude of the estimated coefficients for the ‘dummy policy variables,’ while the vertical axis indicates the density value and the *p*-value. The curves represent the kernel density distribution of the estimated coefficients, the blue dots signify the corresponding *p*-values, and the red vertical dotted line indicates the *p*-value. This paper’s benchmark regression cohort DID model yielded a real estimate of 0.183; the black horizontal dotted line denotes the significance level of 0.1. The figure clearly illustrates that the majority of the estimated coefficient values cluster around 0, with corresponding p-values exceeding 0.1, indicating that most of the estimated coefficients derived from randomly altering the values of the degree of enrollment expansion at the 10 percent level are not statistically significant. Conversely, the actual estimated coefficients derived from the benchmark regression of this study significantly diverge from the majority of the regression outcomes, suggesting that the estimation findings are not coincidental, hence validating the results of the placebo test.

#### 4.3.2 Replacement of variable measurement methods

The assessment of certain critical variables in the benchmark regression may be compromised by measurement inaccuracy. Consequently, this study substitutes the measurement method for these variables to perform robustness tests accordingly.

Initially, this paper substitutes the dependent variables. The Chinese Family Tracking Survey (CFPS) data encompasses statistics regarding individuals’ impressions of their surrounding environment, specifically addressing the survey question, “What is your opinion of the environment in the village or neighborhood where you reside?” Responses about noise pollution, waste disposal, etc., are rated on a scale of 1 to 5, where 1 signifies very good, 2 good, 3 fair, 4 poor, and 5 extremely poor. This paper substitutes the environmental perception variable in the benchmark regression for cohort double-difference analysis, with the regression results presented in column (1) of Table 4, indicating that the revised dependent variables demonstrate that higher education enhances the public’s environmental perception.

**Table 4:** Robustness test: replacing variable measures.

Variable	(1) Replacement of environment-perception variables	(2) Replacement of the expansion level variable	(3) Replacement of cohort measurements
Experience of expansion × degree of expansion	0.082** (0.035)	0.122** (0.050)	0.011** (0.005)
Control variables	Yes	Yes	Yes
Birth cohort fixed effects	Yes	Yes	Yes
Province fixed effects	Yes	Yes	Yes
Observation	8,837	16,043	16,043
*R* ^2^	0.027	0.071	0.071

**denote significance at the 5% level, with standard errors provided in parentheses.

Secondly, this work reconstructs the variables that assess the higher education growth policy. Certain scholars utilize the ratio of students enrolled in local colleges and universities to those in general high schools within each province in 1998 to assess the extent of expansion in their examination of the effects of higher education expansion on family downsizing. Consequently, this paper substitutes the expansion degree variable in the benchmark regression with the variable from the cohort difference-in-difference analysis, and the regression outcomes are presented in Table 4 (2). Column (2) of Table 4 indicates that the results align with those of the benchmark regression, confirming that higher education positively influences public perception of the environment.

This article ultimately reorganizes the fragmented beneficiary birth cohort. This article, adhering to conventional research in benchmark regression, designates a value of 1 to the birth cohort impacted by the college entrance examination expansion policy and a value of 0 to the birth cohort unaffected by the policy. Nonetheless, the birth cohort impacted by the policy of increasing enrollment in the college admission exams may exhibit other disparities within it. Certain scholars have indicated in their research regarding the effects of free compulsory education policies on urbanization that individuals aged 16 and older are unaffected by the policy upon its implementation, whereas those aged 15 benefit from the policy for 1 year, and individuals aged 14 for 2 years, and so forth. This article discusses the following approach: individuals who are 17 years old in the year of the college entrance examination expansion policy implementation are eligible for a one-year extension incentive, while those who are 16 years old can receive a two-year extension incentive, and so forth. This article can reassess the queue for analysis. The regression outcomes of altered queuing measurements are presented in column (3) of Table 4, demonstrating that elevated education substantially enhanced the public’s environmental perception.

#### 4.3.3 Exclusion of other factors

Environmental pollution inflicts both private and public damage, with extensive repercussions. Incorporating it into social security can aid particularly vulnerable groups affected by pollution, thereby contributing to the maintenance of social stability and order. Secondly, residents’ environmental protection behavior exhibits notable characteristics of collective action, while environmental perception serves as the foundation for engaging in such behavior. Additionally, social capital significantly facilitates environmental protection actions (Su et al., 2021). The social security system, as a crucial component of the social framework, may indirectly influence residents’ environmental protection behavior by affecting their social relationships or resource accessibility, thereby impacting the conclusions of this paper. Consequently, to exclude the impact of this variable, this study removes individuals from the sample who are enrolled in basic pension insurance and reassesses the effect of higher education using solely the sample of those not participating in basic pension insurance. The regression findings presented in column (1) of Table 5 demonstrate that the conclusions of this paper are robust.

**Table 5:** Robustness tests: excluding other factors.

Variable	(1) Excluding social security factors	(2) Excluding family planning factors	(3) Excluding family planning factors
Experience of expansion × degree of expansion	0.140** (0.064)	0.570* (0.340)	0.204** (0.084)
Control variables	Yes	Yes	Yes
Birth cohort fixed effects	Yes	Yes	Yes
Province fixed effects	Yes	Yes	Yes
Observation	10,299	1809	6,932
*R* ^2^	0.066	0.071	0.062

*, **denote significance at the 10%, 5% levels respectively, with standard errors provided in parentheses.

A further potentially disruptive policy is the family planning initiative that commenced in 1980, when the Central Committee of the Communist Party of China (CPC) released the ‘Open Letter to All Members of the Communist Party and the Communist Youth League on Controlling China’s Population Growth’ in September 1980, and the ‘one-child-only’ policy was initially proposed at the Third Session of the Fifth National People’s Congress (NPC) in the same year, leading to the extensive implementation of the ‘one-child-only’ policy. Consequently, it may be determined that the ‘one-child policy’ was initially implemented on a significant scale in 1980. Individuals born before September 1981 constituted the primary participants in the 1999 college entrance examination. Given that the expansion of the college entrance examination targeted the entire school-age demographic, it is challenging to entirely isolate the population influenced by the family planning policy but not by the expansion of the college entrance examination from the sample. To eliminate the impact of the family planning policy, this paper emulates the methodologies of existing scholars and employs two approaches to assess the interference policy: firstly, it selects a sample of ethnic minorities for regression estimation, as the ‘one-child’ rule does not apply to these groups, thereby mitigating the influence of the family planning policy. Secondly, samples from Henan, Hebei, Guangxi, Gansu, Inner Mongolia, Yunnan, Qinghai, Ningxia, Hainan, and Xinjiang were chosen for regression estimation, as these regions implemented the ‘two or more children’ birth policy during the initial phase of the family planning policy, thereby eliminating the potential interference of the family planning policy. Columns (2) and (3) of Table 5 demonstrate that the findings of this article remain resilient despite the omission of family planning policies.

## 5 Mechanism testing

This study examines the substantial impact of higher education on public environmental perceptions through benchmark regression, focusing on the implications of the college entrance examination’s expansion at the educational level. Firstly, higher education directly augments the public’s years of schooling; numerous education policies in China fundamentally enhance social equity within the educational sphere. The implementation of free education policies diminishes tuition fees for compulsory education students, thereby facilitating an increase in the enrollment rate of rural children (Xiao et al., 2017). Furthermore, the expansion of higher education fosters the advancement of overall educational equalization of opportunities (Bernardi and Ballarino, 2012). Secondly, the pedagogical model and educational philosophy of higher education facilitate the enhancement of individuals’ proficiency in utilizing instruments for information access, potentially aiding the public in acquiring greater environmental knowledge and subsequently refining their environmental awareness.

Moreover, higher education can augment individual rational thinking among the populace, and the college entrance examination expansion policy distinguishes itself from other educational policies by facilitating the enhancement of comprehensive abilities in underperforming students through knowledge construction inquiry and reflective assessment (Yang et al., 2022). Consequently, higher education is a crucial phase for developing individuals’ character and ethics, serving as a significant procedure for fostering their maturity, which can augment students’ capacity for rational thought. The enhancement of education and individual rational thought elevates citizens’ understanding of environmental difficulties, therefore augmenting their environmental perspective. The enhancement of Internet accessibility augments the efficiency of obtaining environmental information, hence elevating environmental perception.

### 5.1 Increasing the number of years of schooling for individuals

This paper utilizes statistics from the CFPS questionnaire data regarding the question ‘Highest level of education completed?’ as a proxy for years of education, where 0 denotes illiteracy or semi-literacy, and values from 3 to 9 correspond to primary school, junior high school, senior high school, junior college, bachelor’s degree, master’s degree, and doctoral degree, respectively, equating to 6 years for primary education, 9 years for junior high, 12 years for senior high, 15 years for junior college, 16 years for a bachelor’s degree, 19 years for a master’s degree, and 23 years for a doctoral degree. Column (1) of Table 6 experimentally investigates the impact of the tertiary education expansion policy on the public’s years of education. The findings indicate that higher education has markedly augmented the duration of schooling among the populace.

**Table 6:** Mechanism analysis.

Variable	(1) Scientific Knowledge	(2) Access to information 1	(3) Access to information 2	(4) Individual rational thought
Experience of expansion × degree of expansion	0.036** (0.017)	0.104** (0.045)	0.043** (0.014)	−0.073* (0.039)
Control variables	Yes	Yes	Yes	Yes
Birth cohort fixed effects	Yes	Yes	Yes	Yes
Province fixed effects	Yes	Yes	Yes	Yes
Observation	13,052	16,043	16,043	16,043
*R* ^2^	0.286	0.391	0.309	0.059

*, **denote significance at the 10%, 5% levels, respectively, with standard errors provided in parentheses.

### 5.2 Improvement of individual capacity to use the internet to access information

Higher education possesses a degree of sophistication, equipping individuals with scientific instruments that enhance their effectiveness in knowledge retrieval. This paper utilizes the statistics from the CFPS questionnaire regarding the question, ‘How important do you think the Internet is for accessing information?’ as an indicator of individuals’ attitudes toward the Internet, alongside the statistics from the question, ‘Have you used the Internet for learning in the past week?’ The statistics from the inquiry served as an indicator of individual Internet usage. The initial question was rated on a scale of 1–5, with 1 indicating extreme unimportance and 5 indicating extreme importance. The subsequent question was answered with either 0 or 1, signifying non-utilization and utilization for learning, respectively. The regression results presented in columns (2) and (3) of Table 6 demonstrate that tertiary education substantially enhances the public’s opinion of the Internet’s significance as an information access channel, along with the frequency of Internet usage.

### 5.3 Improvement of individual rational thinking

Higher education settings are conducive to the innovation of information literacy education, transforming the conventional pedagogical paradigm and fostering the development of students’ critical thinking (Shahzad et al., 2024). Literature indicates that the educational milieu in higher education is increasingly open and inclusive, progressively diminishing the societal impact of ‘stereotypes’ on individual cognition, which not only fosters inspiration but also enhances the rationalization of students’ thought processes. This article employs the statistics from the CFPS questionnaire regarding ‘trust in strangers’ as a surrogate for the rational thinking variable, with responses ranging from 0 to 10, where higher scores signify increased trust in strangers. The fourth column in Table 6 empirically investigates the impact of higher education on the rational cognition of particular members of the public. The findings indicate that university education renders the public comparatively more cautious toward strangers and enhances rational thought within society, suggesting that tertiary education substantially elevates the level of rational thinking among the populace.

It is also worth noting that, due to data limitations, the scope of explanation for the channel of influence of improving rational thinking is limited, which may be the reason for the low R^2^ value in the regression results in column (4) of Table 6.

## 6 Heterogeneity test

After the aforementioned studies, this research affirms that higher education influences public environmental perceptions and elucidates the process by which this effect is generated; yet, it remains pertinent to further explore the following inquiries: What kind of variation is there in the influence of higher education on public environmental perceptions? Do varying identity groups or areas amplify the influence of higher education on public environmental perceptions due to their inherent differences? This section examines the question of heterogeneity in light of these possibilities.

### 6.1 Gender heterogeneity

This study segregates the sample by gender and conducts separate benchmark regression estimations, with the results presented in the first and second columns of Table 7. The findings indicate that the coefficient values are nearly identical, and both are statistically significant at a minimum of the 5% level. This implies that higher education similarly influences the environmental perceptions of both males and females, revealing no significant gender heterogeneity.

**Table 7:** Heterogeneity test.

Variable	(1) Male	(2) Female	(3) Higher environmental pollution	(4) Minor environmental pollution
Experience of expansion × degree of expansion	0.176** (0.078)	0.175*** (0.065)	0.254*** (0.062)	0.016 (0.095)
Control variables	Yes	Yes	Yes	Yes
Birth cohort fixed effects	Yes	Yes	Yes	Yes
Province fixed effects	Yes	Yes	Yes	Yes
Observation	7,949	8,094	4,321	11,722
*R* ^2^	0.074	0.079	0.072	0.097

**, ***denote significance at the 5, 1% levels, respectively, with standard errors provided in parentheses.

### 6.2 Environmental pollution heterogeneity

Variations in environmental pollution within an individual’s locality can result in disparities in their environmental perceptions. This paper employs the average PM concentration as a criterion to categorize the provinces of the sampled individuals into more and less polluted regions. The findings, presented in columns (3) and (4) of Table 7, indicate that in the more polluted provinces, higher education enhances the public’s environmental perceptions, whereas this effect is not significant in less polluted areas. This indicates that individuals in less environmentally developed regions are more inclined to improve their societal environmental attitudes.

### 6.3 Urban–rural heterogeneity

Despite the overall enhancement of educational access through the implementation of the college entrance examination expansion policy, structural inequality persists. The resource advantages conferred by urban hukou remain pronounced, indicating that barriers to education and opportunities in rural areas, influenced by urban–rural hukou disparities, continue to exist. Consequently, this situation directly affects individuals’ perceptions of their environment regarding higher education. Consequently, this will immediately result in urban–rural disparities regarding the influence of higher education on individuals’ environmental perceptions, i.e., urban–rural heterogeneity. This study analyzes the urban–rural classification of the CFPS questionnaire data utilizing information from the National Bureau of Statistics (NBS), designating the urban sample as 1 and the rural sample as 0. The regression results presented in columns (1) and (2) of Table 8 indicate that the regression coefficients for the urban sample align with the benchmark regression and exhibit high significance, whereas those for the rural sample lack significance.

**Table 8:** Heterogeneity test.

Variable	(1) Towns	(2) Villages	(3) Abundant medical resources	(4) Lack of medical resources	(5) High level of urban sprawl	(6) Low level of urban sprawl
Experience of expansion × degree of expansion	0.254*** (0.067)	0.123 (0.077)	0.173 (0.139)	0.203*** (0.058)	0.201*** (0.069)	−0.117 (0.102)
Control variables	Yes	Yes	Yes	Yes	Yes	Yes
Birth cohort fixed effects	Yes	Yes	Yes	Yes	Yes	Yes
Province fixed effects	Yes	Yes	Yes	Yes	Yes	Yes
Observation	7,763	7,887	3,771	12,272	9,828	5,573
*R* ^2^	0.082	0.071	0.104	0.069	0.074	0.079

***denote significance at the 1% level, with standard errors provided in parentheses.

### 6.4 Heterogeneity of health resources

The availability of regional healthcare resources is intrinsically linked to individual health development. In light of the rising incidence of various illnesses, particularly respiratory diseases attributed to environmental pollution, disparities in healthcare resources may lead to varying degrees of environmental perception among individuals. This paper utilizes the average number of health institutions as a threshold to categorize the provinces of the sample individuals into regions with abundant and limited health care resources. The findings, presented in columns (3) and (4) of Table 8, indicate that in regions with abundant health care resources, the influence of higher education on public environmental perception is negligible, while in regions with limited health care resources, this influence is markedly significant.

### 6.5 Heterogeneity in the extent of urban sprawl

Urban sprawl denotes the low-density extension of urban areas from the city center to the periphery, characterized by leapfrogging and banding, amidst elevated automobile commuting. Scholars like Liu et al. (2023) have demonstrated its considerable adverse effects on regional environments. Urban sprawl signifies the breadth of urban expansion, a phenomenon that frequently detrimentally impacts cities regarding environmental degradation, traffic congestion, resource depletion, and health expenditures, while also influencing individuals’ perceptions of their environment. This work develops an urban sprawl indicator utilizing the subsequent model:


(3) Sprawl=0.5×(L%−H%)+0.5

L% denotes the proportion of the inner city’s light brightness area that is below the national average light level relative to the entire city area, while H% represents the proportion of the inner city’s light data brightness that exceeds the national average level. The urban sprawl degree for each city is computed using Equation 3. Subsequently, the average sprawl degree for the province is derived by aggregating the sprawl degrees of the cities within each province. The average sprawl degree across all provinces serves as a threshold to categorize provinces into high sprawl and low sprawl city provinces. The regression outcomes are presented in columns (5) and (6) of Table 8. The findings indicate that higher education significantly influences public environmental perception in provinces characterized by extensive urban sprawl, whereas this effect is negligible in provinces with minimal urban sprawl. This suggests that urban sprawl contributes to environmental issues, heightening the sensitivity of higher education beneficiaries to these perceptions. This indicates that urban sprawl has generated environmental issues, rendering higher education beneficiaries more attuned to environmental changes, unlike regions where this phenomenon is absent or less pronounced, resulting in a negligible enhancement of public environmental perception.

## 7 Further analysis

A primary objective of environmental regulation is to enhance environmental quality, and the regulatory process serves to mitigate environmental pollution within society. Consequently, there may be a correlation between environmental regulation and shifts in public perception regarding environmental issues as the regulation addresses various environmental challenges. Li and Lv (2021) formulated a dynamic control model to examine the effects of environmental regulation and consumer awareness on the optimal investment strategies of monopolistic innovations. The findings indicate a complementary or substitutive relationship between steady-state investment innovations, implying a potential connection between public environmental perceptions and regional environmental regulations. Gao et al. (2024) demonstrate that environmental rules can substantially influence enterprises’ consciousness regarding environmental protection, which can directly result in varying degrees of environmental pollution generated by firms during production.

This article must analyze the specific roles of environmental regulation in higher education and its impact on public opinion of the environment. This work will categorize environmental regulation into market incentives, command-and-control, and voluntary measures and will interact these categories with the primary explanatory variables of the study, subsequently integrating them into the benchmark regression model (1) for analysis.

### 7.1 Market-incentivized environmental regulation

The sewage fee system in China has a lengthy history, and its implementation process demonstrates the system’s stability, which effectively regulates the pollution behavior of regional enterprises. Consequently, this paper utilizes the province’s sewage fee revenue to assess the level of market incentives for environmental regulation within the province, with the logarithmic processing applied, denoting market incentives for environmental regulation as ERS_1_. The regression outcomes after the incorporation of its interaction term with the principal explanatory variables into model (1) are presented in column (1) of Table 9. These results indicate that the regression coefficients for the market incentive-based environmental regulation and the key explanatory variables are significantly negative, suggesting that market incentive-based environmental regulation can enhance the local environmental pollution levels, thereby exerting a substitution effect in higher education to elevate public environmental perception.

**Table 9:** Further analysis.

Variable	(1) Market-incentivized environmental regulation (*i* = 1)	(2) Command-and-control environmental regulation (*i* = 2)	(3) Voluntary environmental regulation (*i* = 3)
Experience of expansion × degree of expansion × ERS_i_ (*i* = 1, 2, 3)	−0.145** (0.072)	−0.019 (0.089)	0.366** (0.175)
Experience of expansion × degree of expansion	1.579** (0.744)	0.336 (0.697)	0.326*** (0.085)
Control variables	Yes	Yes	Yes
Birth cohort fixed effects	Yes	Yes	Yes
Province fixed effects	Yes	Yes	Yes
Observation	16,043	16,043	16,043
*R* ^2^	0.071	0.071	0.071

**, ***denote significance at the 5%, 1% levels, respectively, with standard errors provided in parentheses.

### 7.2 Command-and-control environmental regulation

Certain scholars, in examining environmental regulation’s impact on green technology innovation, initiate their analysis from the regulation itself to develop a specific metric for its intensity. They utilize the frequency of environmental administrative penalty cases as a representation of ex-post punishment within environmental regulation. This definition aligns with the criteria for command-and-control environmental regulation outlined in this paper; thus, this paper employs the count of environmental administrative penalties in a province as an indicator of the command-and-control regulatory level within that province. Variable indicated as ERS_2_. The regression outcomes after the incorporation of interaction terms with the principal explanatory variables into model (1) are presented in column (2) of Table 9. The findings indicate that command-and-control environmental regulation does not significantly moderate the influence of higher education on the public’s environmental perception.

### 7.3 Voluntary environmental regulation

Certain scholars have categorized environmental regulation within the field of environmental studies, specifically delineating voluntary environmental regulation based on the autonomous actions of the public, utilizing the volume of petitions concerning regional environmental matters to illustrate voluntary environmental regulation. Consequently, utilizing this methodology and accounting for data availability, the quantity of environmental reports within a province was ultimately chosen to quantify the degree of voluntary environmental regulation in that province, denoted as ERS3. The regression outcomes after the incorporation of its interaction term with the principal explanatory variables into model (1) are presented in column (3) of Table 9. This indicates that its coefficient is the most significant in comparison to the other two environmental regulations; however, the positive and negative values of the coefficient contrast with those of the market incentive-based environmental regulation.

This suggests that the voluntary type of environmental regulation facilitates the enhancement of public environmental perception through higher education. This may indicate that voluntary environmental regulation has a limited impact on enhancing environmental standards in comparison to the mandatory measures of command-and-control regulation and is insufficient to generate a substitution effect.

## 8 Conclusion and insights

Global environmental health issues are intensifying, and China is expected to face analogous challenges during its modernization. Consequently, to mitigate resource limitations and environmental stress, the Chinese government has proposed incremental development objectives and associated policies. The country’s environmental changes are intricately linked to the populace’s conduct, and the execution of programs necessitates public collaboration. The success of China’s environmental policies and protection objectives is intricately linked to the evolution of the Chinese populace’s environmental perceptions, which can subsequently transform into environmental concerns or awareness—essential prerequisites for fostering public environmental protection behaviors. Consequently, examining the evolution of public environmental perception and its determinants helps elucidate present environmental attitudes and facilitate the establishment of a conducive ecological environment. The progressive enhancement of public environmental perception is a notable characteristic of the advancement of the Chinese populace in the 21st century. This research aims to connect the significant impact of higher education on individual consciousness and conduct with the enhancement of public environmental perception, serving as an additional explanation for the evolution of environmental behavior among the Chinese populace.

This paper employs data from the 2020 China Family Tracking Survey (CFPS) to construct a difference-in-differences model, leveraging cross-provincial and cohort variations stemming from the 1999 gaokao expansion to rigorously assess the influence of higher education on public environmental perception. The findings indicate that higher education substantially influences public environmental perception, and this fundamental conclusion withstands many robustness assessments, including the parallel trend test and the placebo test, demonstrating considerable robustness.

This study further examines the method by which higher education influences environmental perception, revealing that higher education provides several educational avenues for individuals, hence augmenting their environmental perception. Firstly, it enhances the individual’s educational attainment, providing systematic higher education during formative years, enabling scientific recognition of environmental issues; secondly, the higher education paradigm cultivates a positive disposition toward the Internet, while its educational approach enhances the individual’s capacity to utilize the Internet for information acquisition, thereby significantly increasing the efficiency of understanding environmental information. Moreover, higher education enhances individuals’ critical thinking skills, allowing for a more objective evaluation of environmental situations and thereby fostering increased public awareness of environmental issues. Nonetheless, because to data constraints, the extent of this influence may be restricted, which becomes one of the future objectives of this work. The impact of higher education on public environmental perception varies among different demographics, with a more pronounced effect observed in individuals residing in regions characterized by elevated environmental pollution, urbanization, inadequate healthcare resources, and increased sprawl. Notably, there is no significant gender disparity in this effect. This research also highlights the significant impact of environmental regulation on higher education and its effect on public environmental perspectives. The study indicates that voluntary environmental regulation positively moderates the enhancement of public environmental perceptions through higher education, whereas market-incentivized environmental regulation exhibits a substitutive effect, and command-and-control environmental regulation has no significant impact in this context.

Based on higher education, this study describes the internal mechanism of Chinese residents’ environmental perception enhancement and provides a complementary explanation for the development of Chinese people’s environmental awareness and environmental protection behaviors now and in the future. At the same time, this paper also draws important insights into the current and future urbanization process and the innovation of higher education concepts in China.

First, it improves the shortcomings of past environmental policies and the deficiencies of upcoming environmental policies in the future. Higher education has an influential role in public environmental perception, and continuously improving environmental protection policies is the key to mobilizing citizens to actively cooperate with the implementation of policies and an important way to ensure the sustainable development of China’s ecological environment. Secondly, as the last “training base” for individuals to enter the society, the atmosphere and teaching philosophy of higher education will play a crucial role in the future development of individuals; therefore, the advancement of higher education in the teaching of scientific knowledge should be maintained at all times, and at the same time, the humanistic and caring concept of higher education should be consolidated to ensure that higher education is “people-oriented” and that the public’s perception of the environment is influenced by higher education. Thirdly, the modernization process of rural areas should be promoted with high quality, while the disorderly expansion of urban areas should be reasonably curbed. The research process has found that the lack of economic and medical resources still exists in the rural areas, while the expansion of urban areas has caused a series of environmental problems, so it is necessary to reasonably “neutralize” the two to ensure that the ecological environment of the towns will not continue to deteriorate, as well as to avoid the possible future of the rural areas. The two need to be rationally “neutralized” to ensure that the ecological environment in cities and towns does not continue to deteriorate and to avoid the possible future shortcomings of rural areas. Fourth, pertinent general education courses must be incorporated into higher education reforms to enhance environmental science education and establish a robust scientific foundation for public engagement. Environmental regulation should adopt targeted, tiered measures to reduce information barriers and help form a virtuous cycle of ‘higher education fostering scientific understanding—regulatory optimization encouraging public participation—public participation enhancing regulatory effectiveness.’ Only then will China be able to deepen the process of sustainable development, and the Chinese people will realize that “green water and green mountains are golden mountains.”

Moreover, while this study elucidates the diverse facets and methods via which higher education influences environmental perception, the conclusions derived from cross-sectional data necessitate additional investigation. Consequently, assessing public environmental perception and investigating educational reform from a multi-tiered perspective provide promising avenues for future study enhancement.

## Data Availability

The raw data supporting the conclusions of this article will be made available by the authors, without undue reservation.
